# Neural Substrates for Hand and Shoulder Movement in Healthy Adults: A Functional near Infrared Spectroscopy Study

**DOI:** 10.1007/s10548-023-00972-x

**Published:** 2023-05-18

**Authors:** Julien Bonnal, Canan Ozsancak, Fanny Monnet, Antoine Valery, Fabrice Prieur, Pascal Auzou

**Affiliations:** 1 Service de Neurologie, Centre Hospitalier Universitaire d’Orléans, 14 Avenue de l’Hôpital, 45100 Orleans, France; 2 CIAMS, Université Paris-Saclay, 91405 Orsay Cedex, France; 3 CIAMS, Université d’Orléans, 45067 Orléans, France; 4 Institut Denis Poisson, Bâtiment de mathématiques, Université d’Orléans, CNRS, Université de Tours, Institut Universitaire de France, Rue de Chartres, 45067 Orléans cedex 2, B.P. 6759, France; 5 Département d’Informations Médicales, Centre Hospitalier Universitaire d’Orléans, 14 Avenue de l’Hôpital, 45100 Orleans, France; 6 SAPRéM, Université d’Orléans, Orléans, France

**Keywords:** Cortical activation, Functional near-infrared spectroscopy, Upper limb, Motricity

## Abstract

Characterization of cortical activation patterns during movements in healthy adults may help our understanding of how the injured brain works. Upper limb motor tasks are commonly used to assess impaired motor function and to predict recovery in individuals with neurological disorders such as stroke. This study aimed to explore cortical activation patterns associated with movements of the hand and shoulder using functional near-infrared spectroscopy (fNIRS) and to demonstrate the potential of this technology to distinguish cerebral activation between distal and proximal movements. Twenty healthy, right-handed participants were recruited. Two 10-s motor tasks (right-hand opening-closing and right shoulder abduction-adduction) were performed in a sitting position at a rate of 0.5 Hz in a block paradigm. We measured the variations in oxyhemoglobin (HbO_2_) and deoxyhemoglobin (HbR) concentrations. fNIRS was performed with a 24-channel system (Brite 24®; Artinis) that covered most motor control brain regions bilaterally. Activation was mostly contralateral for both hand and shoulder movements. Activation was more lateral for hand movements and more medial for shoulder movements, as predicted by the classical homunculus representation. Both HbO_2_ and HbR concentrations varied with the activity. Our results showed that fNIRS can distinguish patterns of cortical activity in upper limb movements under ecological conditions. These results suggest that fNIRS can be used to measure spontaneous motor recovery and rehabilitation-induced recovery after brain injury. The trial was restropectively registered on January 20, 2023: NCT05691777 (clinicaltrial.gov).

## Introduction

Stroke is a leading cause of chronic disability worldwide in adults (Johnston et al. 2009; GBD 2019 Stroke Collaborators 2021). Upper extremity (hand and arm) impairments are especially prevalent after stroke and cause lasting disabilities. A study of 102 individuals with upper limb motor deficits showed that only one-third recovered full dexterity at 6 months; lack of recovery markedly reduced autonomy and quality of life (Kwakkel et al. 2003). Clinical and experimental studies have shown that spontaneous recovery occurs to varying extents within days to weeks of the stroke. Functional reorganization of the motor cortex may occur in both the ipsilesional and contralesional hemispheres. If the cortical lesion is small, one recovery mechanism is remapping of the remote ipsilesional primary motor (M1) and ventral premotor cortices (Liepert et al. 2000; Frost et al. 2003; Kato et al. 2020). In non-human primates, focal damage of M1 triggers functional remapping to the adjacent M1, more specifically to the territory formerly occupied by the elbow and shoulder (Nudo and Milliken 1996). A shift of hand-related brain activation to the rim of the infarct has been described in individuals with good post-stroke recovery (Cramer et al. 2006; Carrera et al. 2013). The contralesional motor cortex may play a greater role in recovery when damage is more severe (Kato et al. 2020). Thus, knowledge of brain activation patterns during execution of a movement is important both for neuroscience and neurorehabilitation.

The use of brain imaging techniques for post-stroke follow-up is valuable for understanding the mechanisms of cerebral recovery. Functional MRI (fMRI) enables a precise study of distal upper limb movements; however, it is not used to study proximal movements because it requires strict immobility during the tests. Functional Near-Infrared Spectroscopy (fNIRS) is a non-invasive technique that assesses neural activation through the measurement of cortical oxygenated and deoxygenated hemoglobin concentrations during motor tasks in a natural environment (Udina et al. 2019; Pinti et al. 2020). Low sensitivity to body movement and the system portability make fNIRS suitable for monitoring cortical hemodynamics during most motor tasks. Although it has a poorer spatial resolution than fMRI, it allows the study of motor skills in more ecological conditions. In healthy subjects, several fNIRS studies have investigated upper limb movements, especially the hand (Anwar et al. 2016; Kashou et al. 2016; Csipo et al. 2019; Lee et al. 2019). Few studies have explored wrist (Abtahi et al. 2017; Muthalib et al. 2018) or elbow movements (Delorme et al. 2019). To our knowledge, few fNIRS studies have investigated cortical activation patterns during shoulder and hand movements (Yeo et al. 2013; Yang et al. 2020) and only in healthy individuals. These studies have both reported more important and extensive activation during shoulder movements compared to hand movements. However, these studies also report differences on several points such as unilateral or bilateral activation, the effet of task duration or type of task on the extension of activation.

An important further step in the use of fNIRS in activation studies would be to use this technology in patients with stroke in order to study neuroplastic changes. This implies to have concording methodological approaches and strong data in healthy individuals. Our study aims to contribute to this area of research by further investigating cortical patterns during these upper limb movements in healthy controls.

As remapping of the sensorimotor cortex after stroke with hand impairment can involve the territory of the elbow or shoulder (Nudo and Milliken 1996), we aimed to determine if the cortical activation of these regions (hand and shoulder) could be distinguished by fNIRS recordings in healthy subjects. We hypothesised that the activation pattern observed with fNIRS would differ for shoulder and hand movements. More specifically, we hypothesised that the cerebral activation during hand movements would mainly involve the contralateral hemisphere, particularly the lateral part of the primary motor cortex; whereas activation during shoulder movements would be more medial and more extensive than that of the hand, involving the contralateral premotor region and supplementary motor cortex (Yeo et al. 2013). If these three cortical regions could be distinguished by fNIRS, this technique could be used to measure spontaneous motor recovery and rehabilitation-induced recovery after stroke.

## Materials and Methods

### Participants

Twenty healthy, right-handed individuals (6 males, 14 females; mean (SD) age 30.9 (4.9) years, range 23–40) with no history of neurological, physical, or psychiatric illness were recruited for this study. Four additional individuals were initially recruited, but their data could not be analyzed owing to the poor quality of the fNIRS signal. The Edinburgh Handedness Inventory (Oldfield 1971) was used to evaluate handedness. All subjects had an Edinburgh laterality ratio ≥ 80. All subjects understood the purpose of the study and provided written informed consent prior to participation. This project was approved by the Institutionnal Review Board CPP SUD-EST IV on the June 16, 2020 (no. 2020-A00325-34).

### Experimental Design and Procedure

Participants were asked to sit comfortably in a chair in an upright position during the experiment. They were instructed to practice the two motor tasks several times before the experiments.

**Fig. 1:**
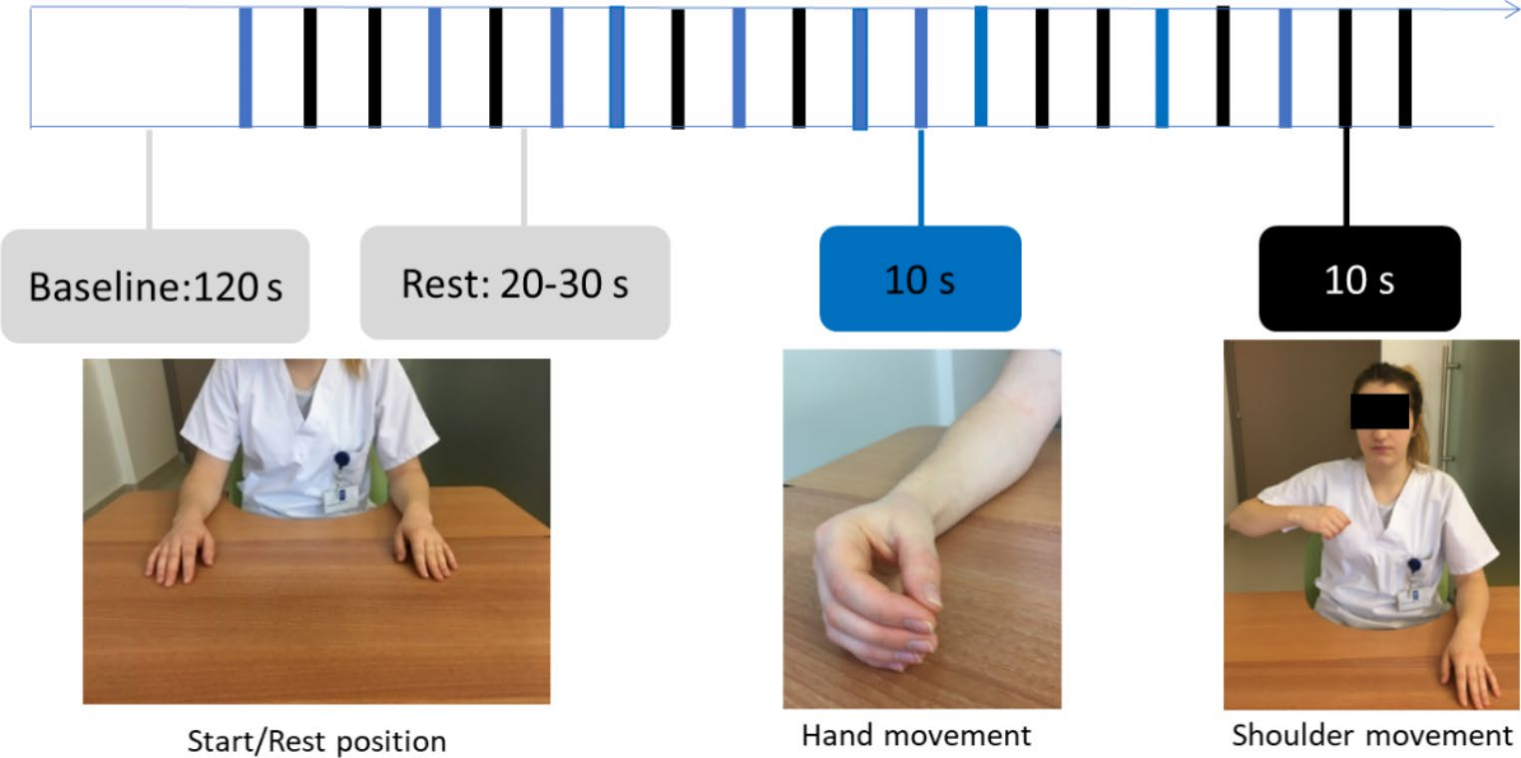
Block design. The blue bars represent hand movements and the black bars represent shoulder movements

The experiments were arranged in a block paradigm (Fig. 1). The block design included 20 trials of 10 s of task. The rest time between trials varied from 20 to 30 s to minimize the physiological effects of breathing, heart rate and Mayer waves (low-frequency arterial pressure oscillations) on the task hemodynamic responses (Leff et al. 2011).

The experiment consisted of two motor tasks:

i. Hand: alternating opening and closing movement of the right hand.
ii. Shoulder: alternating movement of abduction and adduction of the right shoulder with the elbow flexed. The movement began with the upper arm by the person’s side and ended before the trunk began to tilt (range around 70–80 degrees).

All tasks were performed at a frequency of 0.5 Hz, ensured by metronome guidance at a frequency of 1 Hz, corresponding to half of a complete movement. The metronome was switched on during both the motor tasks and rest periods to control for the effects of the auditory stimulus.

The verbal instructions to start the motor tasks were ‘‘Hand” or ‘‘Shoulder’’ for hand, and shoulder movement tasks respectively, and the instruction to stop the motor tasks was ‘‘Stop’’. Participants did not know which motor task was to be performed until the instruction to initiate the motor tasks was given.

The order of experimental sessions was randomized, and the pre-task baseline was 2 min in duration.

### fNIRS Data Acquisition

**Fig. 2:**
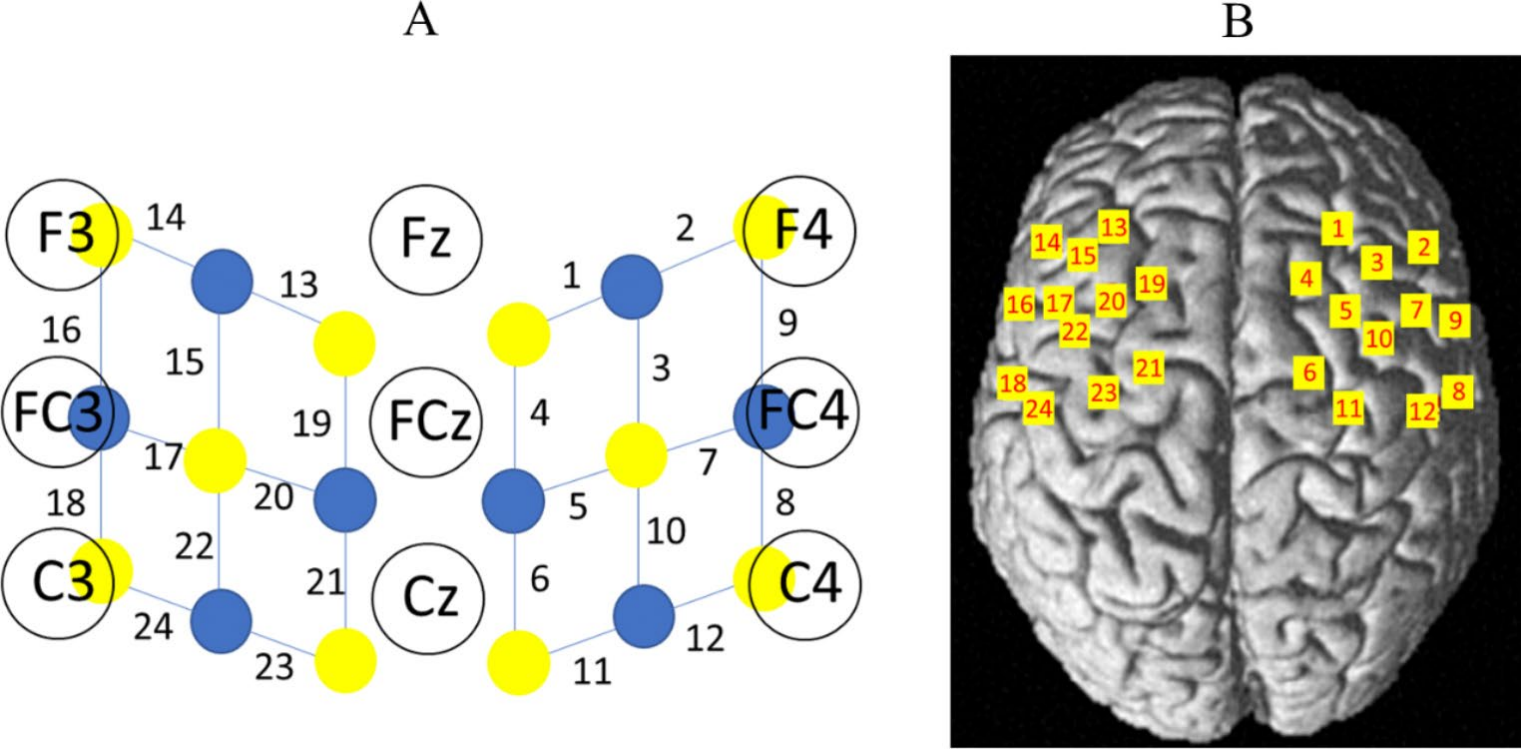
Schematic diagram of the optode locations of the EEG 10/20 system. (**A**) A total of 18 optodes, including 10 light sources (in yellow) and 8 detectors (in blue), were arranged on the scalp to enable 24-channel measurements. (**B**) The anatomical locations of the optodes were superimposed onto the normalized brain surface in the MNI standard brain template

Changes in the concentrations of oxyhemoglobin (HbO_2_) and deoxyhemoglobin (HbR) within the cortex were measured with a continuous wave optical system Brite 24 system (Artinis Medical Systems, Netherlands). The sources of this system generate 2 wavelengths of near-infrared light at 670 and 850 nm, and the sampling rate is fixed at 10 Hz. A total of 10 light sources and 8 detectors with an inter-optode distance of 3 cm constituted 24 channels (Fig. 2A).

To localize the coordinates of each channel in the MNI standard brain (Lancaster et al. 2000), a 3D digitizer (FASTRACK, Polhemus) was used, and the coordinates were further imported to the NIRS SPM (statistical parametric mapping for near-infrared spectroscopy) toolbox for spatial registration (Ye et al. 2009) (Fig. 2B). NIRS SPM is a Matlab toolbox that can be used for processing fNIRS data and projecting the statistical results onto the brain using an anatomical atlas.

### Preprocessing of fNIRS Data

We used both HbO_2_ and HbR signals to measure the hemodynamic response because they provide different and complementary information (Hoshi et al. 2001; Strangman et al. 2002). The Homer2 toolbox in Matlab (The MathWorks Inc.) was used for offline data preprocessing (Huppert et al. 2009).

The processing was as follows:

1. Identification and exclusion of bad channels: channels were considered as bad and excluded from the analysis if the coefficient of variation ([standard deviation/mean]*100) of the raw data was > 33%. The function hmrPruneChannels was used (SNRthresh = 3). The exclusion was done subject by subject. For each one, the number of excluded channels was between 0 and 3 out of 18.
2. Optical density conversion: raw data were converted into optical density with the hmrIntensity2OD function.
3. Identification of motion artifacts: time sections were considered as containing motion artifacts if the signal for any given active channel changed by more than 50 times the standard deviation or by more than 5 standard deviations during a 0.5 s period. The hmrMotionArtifactByChannel function was used (tMotion = 0.5, tMask = 1, STDEVthresh = 50, AMPthresh = 5).
4. Motion artifact correction: sections marked as motion artifacts were corrected with principal component analysis, as movement is the principal source of variance. We used the hmrMotionCorrectPCA function (nSv = 0.8).
5. Physiological artifacts were removed using Principal Component with the enPCAfilter_nSV function.
6. Filtering periodic noise: respiration, cardiac activity and high frequency noise were attenuated with hmrBandpassFilt (hpf = 0, lpf = 0.1).
7. Concentration conversion: corrected optical density data were converted into relative concentration changes with the modified Beer-Lambert law (Kocsis et al. 2006). The age-dependent differential path length factor (DPF) value was calculated for each participant (Scholkman et al., 2010). DPF values were calculated for each wavelength according to the mean age. They were respectively 6.4 and 5.3 for the 760 and 840 nm wavelengths.
8. Hemodynamic response function (HRF) was estimated by solving a general linear deconvolutionmodel (GLM) using the hmrDeconvTB_SS3rd function (t range = [-10, 20], gstd = 1, gms = 1, rhoSD_ssThresh = 1).

### Data Analysis

Data analysis was performed with MATLAB. Mean values were calculated for the rest (from 10 s before, to the beginning of the task) and trial periods (from + 5 s to + 15 s) for each channel. To detect cerebral activation, the mean changes in HbO_2_ and HbR between the rest period and condition for each channel were compared using the Student t-test. For each condition, we performed 24 t-tests with a significance level set at p < 0.05. To control for the growth of the false discovery rate (FDR) due to multiple comparisons, we employed the Benjamini-Hochberg procedure (Benjamini and Hochberg 1995). For task comparisons, we analyzed the mean changes in HbO2 and HbR concentrations between the two conditions for each channel using one-way repeated measures ANOVA. Post-hoc analysis was conducted using paired Student’s t-tests. A total of 24 t-tests were performed for each condition or comparison, with a significance level set at p < 0.05. (Bonferroni correction).

## Results

Results for cerebral activation and task comparisons are shown in Table 1.

### Comparison of Baseline and Task Hemodynamic Responses: Cerebral Activation

**Fig. 3:**
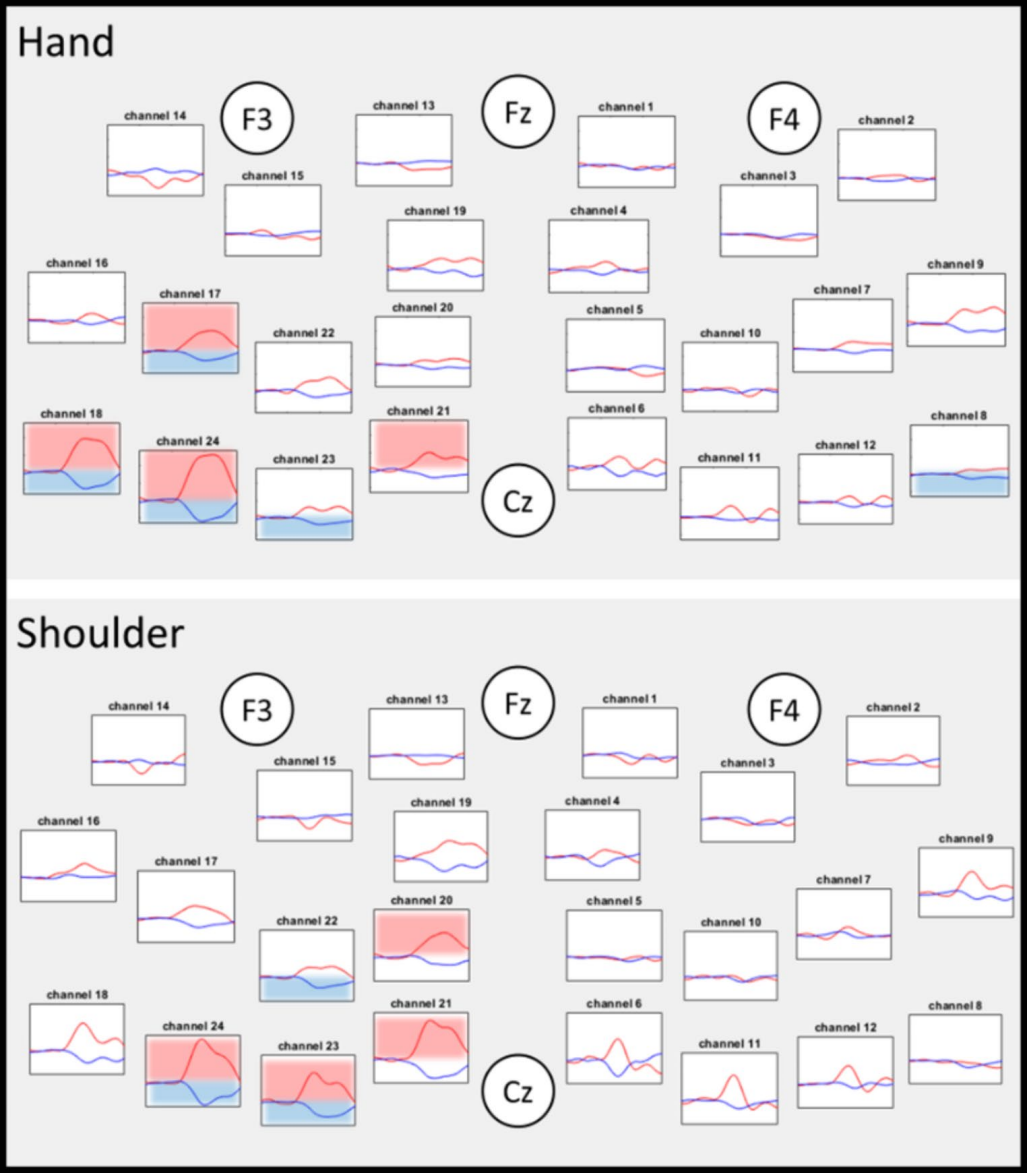
Results of the hemodynamic response by task (Hand and Shoulder) for each channel. The results are expressed as means (average of the participants). Graph locations were organized according to the anatomical correspondence using the EEG 10/20 system. The time window analyzed was 30 s: from 10 s before the beginning of the task to 20 s after. The red traces indicate HbO_2_ concentrations and the blue traces indicate HbR concentrations. The red boxes indicate a significant difference between rest and task periods for HbO_2_ concentration. The blue boxes indicate a significant difference between rest and task periods for HbR concentrations. p < 0.05 FDR corrected

**Fig. 4:**
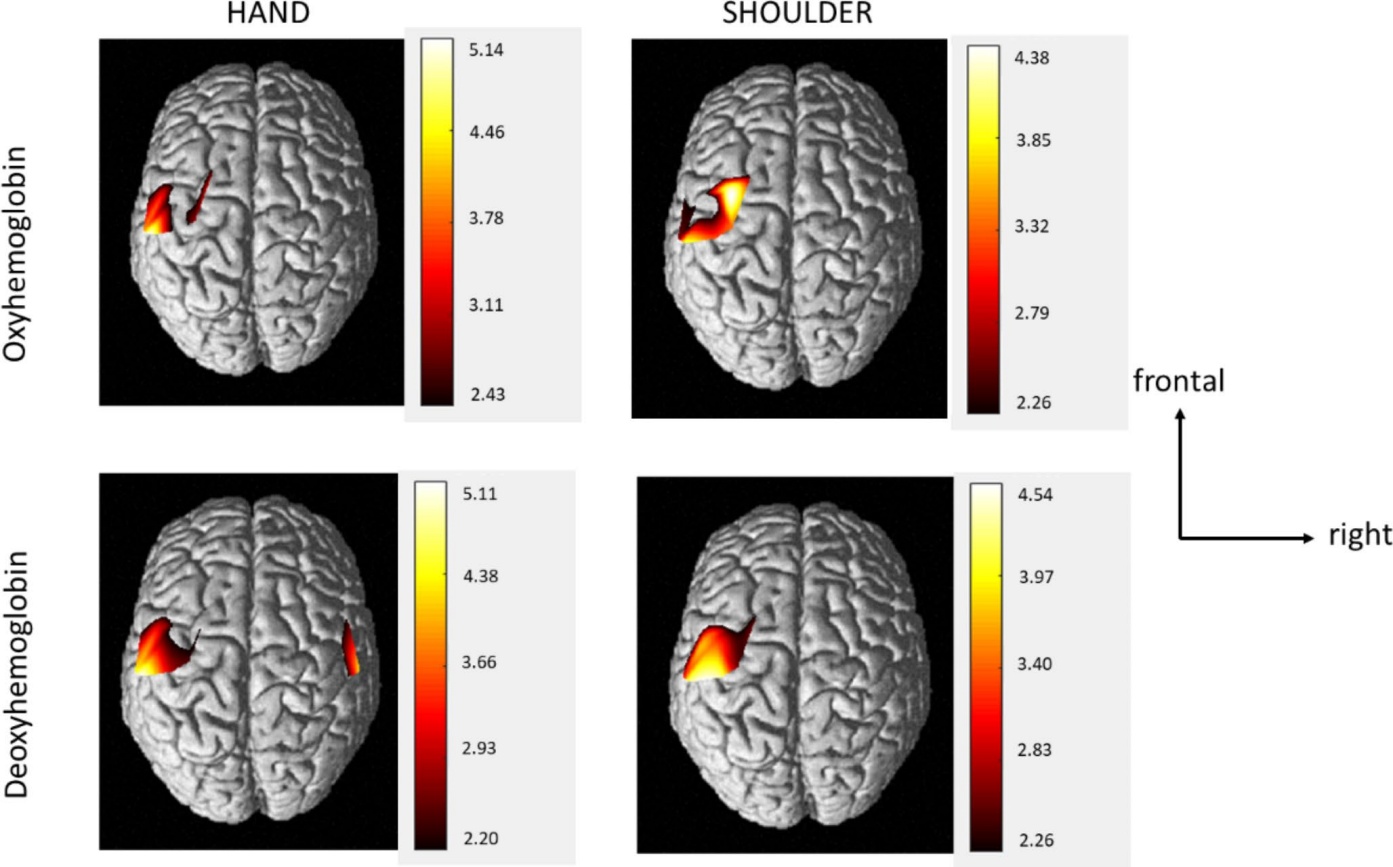
Mean cerebral cortex activation maps for HbO_2_ and HbR during the hand and shoulder tasks. Data are *t* values, *t*: statistical value of sample *t*-test with a significance level of p < 0.05 ( FDR corrected). The change from red to yellow indicates that the degree of activation is from low to high. Only statistically significant responses are illustrated. The data and maps were calculated and generated by NIRS-SPM

The hemodynamic responses for both conditions are illustrated by the plotogramms (Fig. 3) and a NIRS-SPM representation (Fig. 4). Overall, the responses were canonical with an increase in HbO_2_ concentration and a tendency towards a decrease in HbR concentration.

For the hand and shoulder tasks, common brain regions showed a significant increase in HbO_2_ concentration, a significant decrease in HbR concentration, or both. The regions involved were contralateral to the task: primary motor cortex (CH21, CH23) and somatosensory cortex (CH24). We also found specific activation for each task. The hand movement resulted in an activation in premotor cortex (CH17) and somatosensory cortex (CH18) and the shoulder movement resulted in an activation in premotor cortex (CH20) and primary motor cortex(CH23).

For both tasks, the activated areas were mainly contralateral. However we also found anipsilateral activation in the somatosensory cortex (CH8) only for the hand task.

**Table 1:** Changes in HbO_2_ and HbR concentrations and the corresponding p-values between rest and task for each condition and between the tasks

CH	coordinates (Brodmann areas)	HbO_2_						HbR					
		Hand		Shoulder		Hand versus shoulder	Shoulder versus hand	Hand		Shoulder		Hand versus shoulder	Shoulder versus hand
		Changes in HbO_2_	p-value	Changes in HbO_2_	p-value	p-value	p-value	Changes in HbR	p-value	Changes in HbR	p-value	p-value	p-value
1	Includes Frontal eye fields	-0.017 ± 0.051	0.928	-0.021 ± 0.079	0.874	0.428	0.572	-0.018 ± 0.052	0.066	-0.018 ± 0.051	0.063	0.492	0.508
2	Dorsolateral prefrontal cortex	-0.006 ± 0.061	0.370	0.019 ± 0.128	0.261	0.705	0.295	-0.007 ± 0.029	0.153	-0.008 ± 0.060	0.285	0.528	0.472
3	Includes Frontal eye fields	-0.030 ± 0.081	0.987	-0.017 ± 0.117	0.742	0.650	0.350	-0.012 ± 0.040	0.097	-0.025 ± 0.071	0.066	0.748	0.252
4	Pre-Motor and Supplementary Motor Cortex	0.021 ± 0.098	0.154	0.021 ± 0.133	0.241	0.508	0.492	-0.010 ± 0.046	0.181	-0.027 ± 0.101	0.125	0.799	0.201
5	Pre-Motor and Supplementary Motor Cortex	-0.017 ± 0.047	0.933	-0.004 ± 0.079	0.592	0.731	0.269	0.005 ± 0.034	0.741	-0.013 ± 0.045	0.111	0.953	0.047
6	Pre-Motor and Supplementary Motor Cortex	0.022 ± 0.142	0.250	0.013 ± 0.086	0.249	0.393	0.607	-0.033 ± 0.117	0.110	-0.034 ± 0.106	0.083	0.514	0.486
7	Pre-Motor and Supplementary Motor Cortex	0.037 ± 0.083	0.028	0.019 ± 0.146	0.281	0.295	0.705	-0.014 ± 0.038	0.056	-0.002 ± 0.067	0.436	0.215	0.785
8	Primary Somatosensory Cortex	0.023 ± 0.072	0.084	-0.013 ± 0.096	0.718	0.055	0.945	-0.020 ± 0.021	< 0.001*	-0.025 ± 0.055	0.027	0.663	0.337
9	Pre-Motor and Supplementary Motor Cortex	0.073 ± 0.137	0.014	0.062 ± 0.133	0.025	0.381	0.619	-0.040 ± 0.071	0.010	-0.017 ± 0.075	0.166	0.107	0.893
10	Pre-Motor and Supplementary Motor Cortex	-0.017 ± 0.065	0.876	-0.010 ± 0.075	0.721	0.658	0.342	-0.007 ± 0.023	0.098	-0.012 ± 0.046	0.132	0.704	0.296
11	Primary Motor Cortex	0.011 ± 0.120	0.347	0.027 ± 0.010	0.131	0.665	0.335	-0.012 ± 0.044	0.122	-0.027 ± 0.058	0.025	0.836	0.164
12	Primary Somatosensory Cortex	0.009 ± 0.145	0.391	0.018 ± 0.111	0.241	0.592	0.408	-0.019 ± 0.062	0.091	-0.006 ± 0.076	0.355	0.236	0.764
13	Includes Frontal eye fields	-0.032 ± 0.103	0.972	-0.038 ± 0.080	0.977	0.359	0.641	0.012 ± 0.030	0.955	0.001 ± 0.041	0.560	0.881	0.119
14	Dorsolateral prefrontal cortex	-0.051 ± 0.090	0.990	-0.025 ± 0.137	0.784	0.800	0.201	0.014 ± 0.032	0.970	-0.004 ± 0.074	0.417	0.910	0.090
15	Dorsolateral prefrontal cortex	-0.019 ± 0.068	0.890	-0.023 ± 0.111	0.822	0.434	0.566	-0.002 ± 0.034	0.394	0.005 ± 0.042	0.698	0.277	0.723
16	Pre-Motor and Supplementary Motor Cortex	0.027 ± 0.092	0.097	0.050 ± 0.109	0.026	0.767	0.233	-0.012 ± 0.039	0.091	0.016 ± 0.044	0.724	0.056	0.944
17	Pre-Motor and Supplementary Motor Cortex	0.108 ± 0.162	0.004*	0.049 ± 0.111	0.031	0.044	0.956	-0.047 ± 0.066	0.003*	-0.035 ± 0.049	0.002	0.253	0.747
18	Primary Somatosensory Cortex	0.176 ± 0.194	< 0.001 *	0.081 ± 0.136	0.008	0.027	0.973	-0.082 ± 0.083	< 0.001*	-0.043 ± 0.061	0.003	0.019	0.981
19	Pre-Motor and Supplementary Motor Cortex	0.051 ± 0.084	0.007	0.074 ± 0.131	0.011	0.776	0.224	-0.012 ± 0.056	0.062	-0.046 ± 0.087	0.015	0.863	0.137
20	Pre-Motor and Supplementary Motor Cortex	0.027 ± 0.113	0.145	0.098 ± 0.106	< 0.001 *	0.996	0.004*	-0.019 ± 0.061	0.087	-0.029 ± 0.064	0.027	0.689	0.311
21	Primary Motor Cortex	0.080 ± 0.119	0.003*	0.163 ± 0.215	0.002*	0.955	0.045	-0.037 ± 0.066	0.012	-0.080 ± 0.138	0.009	0.925	0.085
22	Pre-Motor and Supplementary Motor Cortex	0.064 ± 0.141	0.028	0.047 ± 0.121	0.049	0.287	0.713	-0.031 ± 0.061	0.017	-0.042 ± 0.057	0.002*	0.699	0.301
23	Primary Motor Cortex	0.051 ± 0.116	0.031	0.108 ± 0.145	0.002*	0.969	0.031	-0.033 ± 0.050	0.004*	-0.062 ± 0.064	< 0.001*	0.971	0.029
24	Primary Somatosensory Cortex	0.237 ± 0.207	< 0.001 *	0.166 ± 0.187	< 0.001 *	0.067	0.933	-0.012 ± 0.069	< 0.001*	-0.082 ± 0.085	< 0.001*	0.224	0.776

*: p < 0.05 FDR corrected

Data are mean ± SEM, unit: µMol/L; channels 1 to 12 correspond to the right hemisphere and channels 13 to 24 correspond to the left hemisphere

Abbreviations: CH = channel; HbO_2_ = oxyhemoglobin; HbR = desoxyhemoglobin.

### Comparison of Hemodynamic Responses Between Hand and Shoulder Tasks

A one-way ANOVA for HbO showed a significant task effect only for CH20 (F = 8.49; p = 0.009). Post-hoc analysis revealed a superiority for the shoulder task (p = 0.004). Similarly, for HbR, a one-way ANOVA indicated a significant task effect only for CH18 (F = 5; p = 0.038), with post-hoc analysis showing a superiority for the hand task (p = 0.019).

## Discussion

This fNIRS study of the hemodynamic response during hand and shoulder movements in healthy subjects confirmed our hypotheses that hand movement is associated with contralateral hemisphere activation in the region of the sensorimotor cortex, and shoulder movement is associated with more medial activation than hand movement. However, in contrast with one of our hypotheses, we did not observe more extensive activation towards the more anterior regions during shoulder movement. These results suggest that fNIRS is a useful alternative to fMRI for the evaluation of proximal limb movement and could be used to investigate cerebral activation in individuals with brain injury.

Numerous studies have provided precise information on the neurological control of the hand in animals and humans (Azim and Alstermark 2015; Boraud et al. 2018; Bruurmijn et al. 2021; Sobinov and Bensmaia 2021). We studied simple movements of the right hand (opening - closing) in right-handed, healthy individuals. We mainly observed lateral activation within the left hemisphere. This result is consistent with the conventional view that voluntary movements derive primarily from cortical hemisphere activation contralateral to the moving limb. For simple motor tasks, hemodynamic changes have been shown to be maximal over the contralateral cortex for channels centered on the motor cortex (M1) (Kim et al. 1993; Maki et al. 1995; (Obrig et al. 1996a, b; Watanabe et al. 1996; Hirth et al. 1997; Strangman et al. 2003; Durduran et al. 2004; Sato et al. 2006; Holper et al. 2009; Lee et al. 2019). This is particularly true for simple movements performed with the right, dominant hand (Sobinov and Bensmaia 2021).

Hemodynamic changes have also been described in the ipsilateral supplementary motor cortex (Obrig et al. 1996a; Wriessnegger et al. 2008; Derosière et al. 2014) suggesting the ipsilateral hemisphere plays an active and specific role in interhemispheric inhibition and facilitation and the planning and execution of voluntary movements (Bundy et al. 2018; Bundy and Leuthardt 2019; Bruurmijn et al. 2021). However, ipsilateral changes are inconsistant (Durduran et al. 2004). The region of the motor cortex activated during ipsilateral hand movements is spatially distinct from that activated during contralateral hand movements (Cramer et al. 2006). In our study, the change in ipsilateral activation between baseline and task was weak compared to contralateral activation. Indeed, for the hand task, only one ipsilateral channel showed activity in HbR. However, several ipsilateral channels exhibited p-values < 0.05, but did not pass the statistical correction threshold.This could be explained by the fact that the participants performed simple tasks with their right, dominant hand whereas ipsilateral responses are stronger for the non-dominant hand (Kim et al. 1993; Lee et al. 2019) or during complex tasks (Verstynen et al. 2005; Anwar et al. 2016).

To our knowledge, only two fNIRS studies compared hand and shoulder movements (Yeo et al. 2013; Yang et al. 2020). Yeo et al. compared brain activation between movements of the right hand and shoulder in nine healthy controls (Yeo et al. 2013). They measured changes in HbO_2_ and total hemoglobin (sum of HbO_2_ and HbR) concentrations in the left hemisphere in three main regions: SM1 (primary sensory motor cortex), PMC (premotor cortex) and PFC (prefrontal cortex). They did not report HbR concentrations. During movements of the right hand, activation was found only in the the left SM1. These results are similar to our findings, also for right hand movements. By contrast, movements of the right shoulder generated a stronger response in the three regions than the hand task. Their interpretation of this difference was that shoulder movements require greater neural recruitment than hand movements. It has been suggested that proximal joint muscles are partly controlled by the corticoreticulospinal tract, which originates mainly from the premotor cortex (PMC) (Jang and Lee 2019). Although they did not directly statistically compare each channel, the authors found more medial activation for shoulder movements and more lateral activation for hand movements, similarly to our study. According to the classical homunculus, the area representing the shoulder muscles is more medial than that of the finger muscles in the primary motor cortex (Schott 1993).

In a more recent study, 20 healthy, right-handed individuals performed similar hand and shoulder tasks to those in our study (Yang et al. 2020) during a bilateral recording of HbO_2_ and HbR concentrations. In their 10-s duration hand task, the authors observed changes in the same regions as we did in our study. However, the variations only concerned HbO_2_ concentration. Instead, we observed a canonical response with an increase in HbO_2_ concentration and a decrease in HbR concentration in most activated regions. For shoulder movements, they found a more extensive cortical response, concerning both the contralateral medial regions and the ipsilateral motor regions.

Together with the results of those two studies, our results indicate that fNIRS can distinguish between hand and shoulder movements. In all three studies, hand movements were associated with low levels of contralateral activation in the lateral motor regions. The results were less consistent for shoulder movements. Yeo et al. found a marked medial and anterior extension towards the premotor regions, which we did not. Their study does not provide information on right hemisphere activation because the optodes were only placed on the left hemisphere. Unlike our results, which showed exclusive contralateral medial activation, Yang et al. found both contralateral and ipsilateral activation. An fMRI study in 11 healthy subjects studied brain activation during hand, elbow, shoulder, hip and ankle movements (Kocak et al. 2009). For hand and shoulder movements, they found similar results to ours with more lateral activation for the hand and more medial activation for the shoulder. Although the spatial resolution of the fNIRS is not as good as that of fMRI, it is sufficiently precise to distinguish specific activation patterns. Moreover, fNIRS can be used in a more ecological situation than fMRI to explore human movements.

fMRI and fNIRS are both based on the physiological principles of neurovascular coupling, the process by which active brain regions induce a local increase in blood flow to match their energy demands via the dilation of capillaries and arterioles (Mishra 2017). fMRI measures the blood oxygen level-dependent (BOLD) response corresponding to the ratio of oxy to deoxy-hemoglobin (Chen et al. 2020). However, the two types of hemoglobin are not individually measured with fMRI. In contrast, fNIRS measures these two types of hemoglobin separately. During neurovascular coupling, the amount of oxygen supplied is typically greater than that consumed locally, resulting in a substantial increase in HbO_2_ concentration and a slight reduction in HbR concentration in the region. These typical changes in HbO_2_ and HbR concentrations are called the canonical hemodynamic response.

There is a debate as to how to interpret variations in the concentration of each chromophore when the canonical response is not observed. If we take fMRI as the gold standard, HbR would theorically be the most reliable parameter reflecting activation. However, few studies have found strong correlations between HbR concentration and fMRI BOLD signals. HbO_2_ concentration seems to be more sensitive to brain activation than HbR (Kleinschmidt et al. 1996; Toronov et al. 2001, 2003; Strangman et al. 2002; Huppert et al. 2006; Zama and Shimada 2015; Hoshi 2016; Nishiyori et al. 2016; Gentile et al. 2019) but is more sensitive to artefacts. The HbR response is more spatially localized (i.e. stimulus evoked HbR concentration decreases in only a few channels), whereas that of HbO_2_ is more generalized, with typical responses being observed in almost all NIRS channels (Hirth et al. 1997; Cannestra et al. 2003; Sato et al. 2007; Dravida et al. 2018). Additionally, decreases in HbR concentration are not homogenous across individuals (Maki et al. 1996; Miyai et al. 2001), and statistically significant changes in HbR concentration do not occur in all individuals (Watanabe et al. 1996). There are discrepancies between studies regarding the type of response observed. For instance, in the study by Yang et al., with a task comparable to ours, finger movements only induced variations in HbO_2_ concentration. In our study, we found two types of variation in the same regions: an increase in HbO_2_ concentration, or a complete canonical response. In the shoulder task, Yang et al. mostly found variations in HbR concentration in the regions where we observed an increase in HbO_2_ concentration, a decrease in HbR concentration or a complete canonical response. Therefore, each hemoglobin type may have advantages regarding the detection of cerebral activation in different motor paradigms. Analysis and reporting of all the available hemoglobin data in fNIRS is recommended to better understand the task-evoked cortical activation patterns (Chen et al. 2020).

This study has several limitations. The first relates to preprocessing quality. The use of short channels is recommended for the preprocessing of fNIRS data (Yucel et al. 2021) but they were not available for this study. Our study has some limitations related to recruitment. First, we only recruited right-handed participants and simple motor tasks were performed on the dominant side. Therefore, our results cannot be extrapolated to the non-dominant side, left-handed individuals or complex motor tasks. Secondly, we only recruited young subjects, whereas some studies have shown that cortical activation patterns are different in older individuals (Berger et al. 2020; Yuan et al. 2022). For instance, a study comparing brain activation between young and elderly healthy subjects during a grasping task found greater activation in the elderly (Berger et al. 2020). Another study showed that activation was more bilateral in older subjects during a hand rehabilitation exercise using a multisensory glove (Yuan et al. 2022). Therefore, our results cannot be directly generalized to elderly people or stroke patients, who are older than our study participants, justifying further studies with elderly subjects.

## Conclusion

Characterization of cortical activation patterns during movements in healthy adults increases understanding of how the injured brain functions. Our findings support and extend the motor control literature on upper limb motor control. Unilateral movements require essentially contralateral activation of the sensorimotor cortex Hand activity is mainly driven by neuronal activation in a limited part of the contralateral lateral sensorimotor cortex. Movements of the shoulder require more medial activation than movements of the hand. These activation patterns can also be influenced by age and laterality. Therefore, future studies should investigate the impact of these parameters on activation patterns during motor tasks.This study confirms the value and feasibility of using fNIRS to understand normal motor control. The fNIRS measures distinguished between proximal and distal tasks and between brain regions; thus, this technique could be used to measure spontaneous motor recovery and rehabilitation-induced recovery after brain injury.

Finally, we found changes in both HbO_2_ and HbR concentrations; therefore, we recommend that future research includes an analysis and report of all the available hemoglobin data from fNIRS to increase understanding of task-evoked cortical activation patterns.
